# Ultrasonography for proper endotracheal tube placement confirmation in out-of-hospital cardiac arrest patients: two-center experience

**DOI:** 10.1186/2036-7902-6-S1-A29

**Published:** 2014-01-31

**Authors:** Jen-Tang Sun, Shyh-Shyong Sim, Hao-Chang Chou, Kah-Meng Chong, Matthew Huei-Ming Ma, Wan-Ching Lien

**Affiliations:** 1 Department of Emergency Medicine, Far Eastern Memorial Hospital, New Taipei City, Taiwan; 2 Department of Emergency Medicine, National Taiwan University Hospital, Taipei City, Taiwan

## Background

A secure airway and effective ventilation are key components of advanced life support [1]. Unrecognized misplacement of endotracheal tube can lead to morbidity and mortality, with a reported incidence of approximately 2.9–16.7% in previous cardiac arrest studies [2–4]. Many traditional methods, including direct visualization of the vocal cords, observation of chest expansion, and chest auscultation can be employed to confirm endotracheal tube position, but each of these methods has limitations and may interrupt chest compressions during cardiopulmonary resuscitation (CPR)[5,6]. Quantitative waveform capnography is recommended as the gold standard for confirming correct endotracheal tube placement in the 2010 American Heart Association (AHA) Guidelines for CPR and Emergency Cardiovascular Care (ECC) [1]. However, it has well-known limitations in cardiac arrest patients, and can be affected by low cardiac output, low pulmonary flow, airway obstruction, or epinephrine use [6,7]. Ultrasonography is a non-invasive, real-time diagnostic tool commonly used during resuscitation. Real-time airway sonographic approaches could enhance physician confidence and decision-making in relation to tracheal tube placement, and may have a role in combination with continuous capnography in emergency patients [8-10].

## Objective

This study aims to evaluate the accuracy of tracheal ultrasonography for assessing endotracheal tube position in cardiac arrest patients.

## Patients and methods

We performed a prospective two-center observational study for adult patients with cardiac arrest undergoing emergency intubation during CPR. Real-time tracheal ultrasonography was performed during the intubation with the transducer placed transversely just above the suprasternal notch, to assess for endotracheal tube positioning and exclude esophageal intubation. The position of trachea was identified by a hyperechoic air-mucosa (A-M) interface with posterior reverberation artefact (comet-tail artefact). The endotracheal tube position was defined as endotracheal if single A-M interface with comet-tail artefact was observed. Endotracheal tube position was defined as intra-esophageal if a second A-M interface appeared, suggesting a false second airway (double tract sign). The gold standard of correct endotracheal intubation was the combination of clinical auscultation and quantitative waveform capnography. The main outcome was the accuracy of tracheal ultrasonography in assessing endotracheal tube position during CPR.

## Results

Among the 96 patients enrolled, 7 (7.3%) had esophageal intubations. The sensitivity, specificity, positive predictive value, and negative predictive value of tracheal ultrasonography were 98.9% (95% confidence interval [CI]: 94.0-99.8%), 100% (95% CI: 61-100.0%), 100% (95% CI: 95.9-100.0%) and 85.7% (95% CI: 48.7-97.4%), respectively. Positive and negative likelihood ratios were 7.0 (95% CI: 1.1-43.0) and 0.0, respectively.

## Conclusion

Real-time tracheal ultrasonography is an accurate method for identifying endotracheal tube position during CPR without the need for interruption of chest compression.

